# Supplementary material for: Silicon Supply Improves Leaf Gas Exchange, Antioxidant Defense System and Growth in Saccharum officinarum Responsive to Water Limitation
Source: Plants (Basel). 2020 Aug 14;9(8):1032. doi: 10.3390/plants9081032 (PMC7464948; doi:10.3390/plants9081032)
Supplement: Supplementary file 1 [file plants-09-01032-s001.pdf]

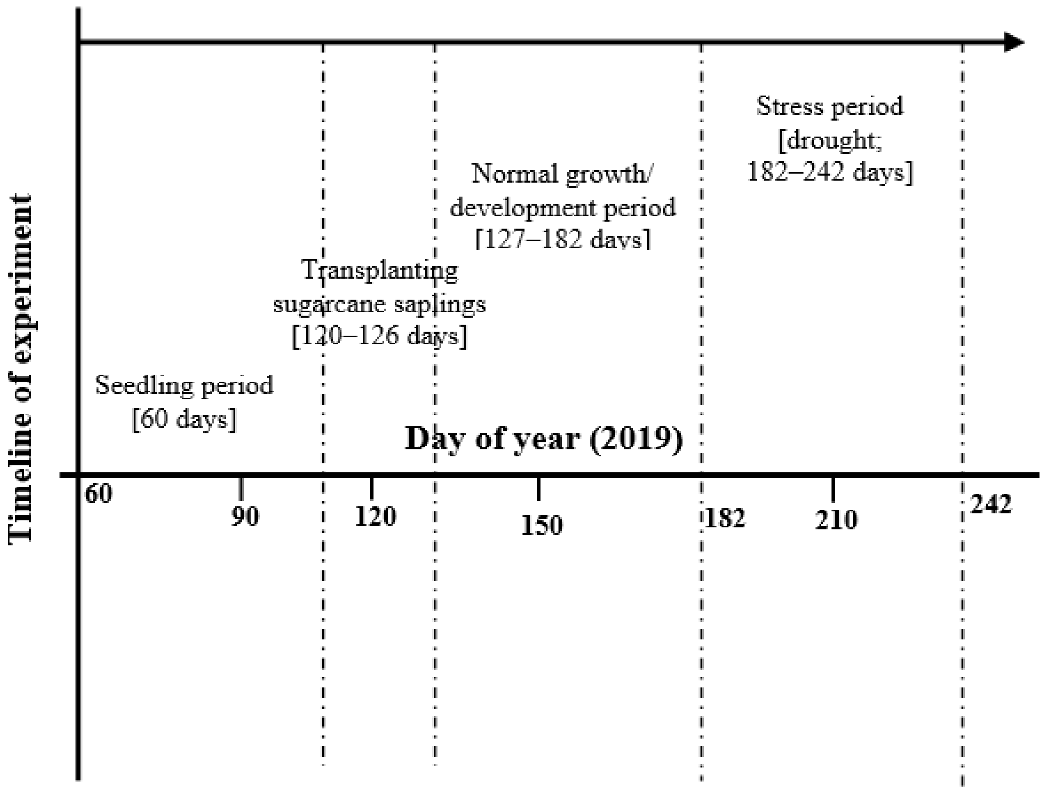

Figure S1. Outline indicates the days of plants period of the whole experiment.

Table S1. Soil properties in the study. Values are shown as the mean.

Soil Characteristics	Unit	Value
Coarse sand	%	16.1
Fine sand	%	42.0
Silt	%	19.9
Clay	%	23.9
pH	-	5.89
Organic carbon	%	0.69
Total N	%	0.11
Available P	mg kg^{-1}	9.08
Available S	mg kg^{-1}	11.95
Exchangeable K	cmol(+)kg^{-1}	2.68
Exchangeable Ca	cmol(+)kg^{-1}	3.8
Exchangeable Mg	cmol(+)kg^{-1}	1.5
Exchangeable Na	cmol(+)kg^{-1}	0.079
Available Si	mg kg^{-1}	85.9
Available Cu	mg kg^{-1}	0.87
Available Fe	mg kg^{-1}	11.9
Available Zn	mg kg^{-1}	1.29
Available Mn	mg kg^{-1}	17.3
Available B	mg kg^{-1}	0.09
Exchangeable Al	cmol(+)kg^{-1}	0.005
Cation exchange capacity	cmol(+)kg^{-1}	9.7

Table S2. Model parameters of morpho-physiological and antioxidant enzymes in responsive to different soil moisture levels with silicon.

Character	SML [%]	α	β	λ	ω	r	S
Plant length [cm]	100-95	−3.428	1.155	−1.026	1.027	0.645	48.015
	80-75	−4.113	6.981	−5.948	1.095	0.715	35.104
	55-50	−3.650	4.517	−3.959	1.035	0.666	20.320
	35-30	−3.952	2.991	−2.559	1.056	0.672	15.923
Leaf number	100-95	−2.659	2.881	−1.066	1.226	0.525	1.648
	80-75	−3.389	1.712	1.257	9.540	0.990	0.305
	55-50	2.628	1.262	9.159	8.442	0.920	0.726
	35-30	−2.357	1.158	6.922	5.460	0.987	0.246
Leaf area-expansion [m^2 $plant^{-1}$]	100-95	2.335	1.051	7.625	7.772	0.747	0.012
	80-75	−1.385	3.307	6.962	3.177	0.986	0.003
	55-50	3.054	5.487	5.163	8.478	0.384	0.016
	35-30	−3.401	1.441	3.821	8.453	0.974	0.004
Leaf water content [%]	100-95	−2.982	3.663	8.939	9.746	0.755	0.081
	80-75	−3.435	1.923	7.792	8.873	0.998	0.143
	55-50	2.639	3.204	6.624	7.634	0.964	1.197
	35-30	1.615	1.098	4.176	9.412	0.263	1.689
Photosynthetic CO_2 assimilation rate [A; μmol CO_2 m^{-2} s^{-1}]	100-95	1.888	5.233	2.487	1.106	0.729	0.582
	80-75	−8.264	1.295	1.034	1.819	0.974	0.601
	55-50	2.547	3.237	1.630	8.484	0.991	0.556
	35-30	5.908	−2.544	1.150	7.150	0.999	0.102
Stomatal conductance (gs; mmol H_2O m^{-2} s^{-1})	100-95	−1.272	−3.278	1.733	1.185	0.983	7.950
	80-75	−1.397	−8.768	1.078	1.064	0.982	6.519
	55-50	2.643	2.013	9.813	7.945	0.992	3.533
	35-30	−1.225	−2.094	7.707	1.177	0.989	4.133
Transpiration rate [E; mmol H_2O m^{-2} s^{-1}]	100-95	2.047	5.861	2.911	1.023	0.921	0.309
	80-75	2.267	2.454	2.059	9.522	0.970	0.080
	55-50	8.870	3.991	1.656	7.163	0.978	0.112
	35-30	2.687	3.145	1.322	7.096	0.951	0.136
Leaf greenness [SPAD value; units]	100-95	8.638	7.564	4.433	1.722	0.998	0.043
	80-75	−2.714	1.546	4.067	6.530	0.996	0.178
	55-50	6.073	−1.037	3.568	7.090	0.987	0.225
	35-30	1.632	5.609	3.041	9.409	0.292	1.110
Chlorophyll a [Chl a; mg g^{-1} FW]	100-95	5.581	−1.843	1.406	1.032	0.379	0.051
	80-75	1.954	1.962	1.267	9.318	0.188	0.061
	55-50	1.209	−4.467	1.090	7.162	0.995	0.006
	35-30	−1.361	3.638	3.820	3.561	0.870	0.010
Chlorophyll b [Chl b; mg g^{-1} FW]	100-95	2.584	3.257	4.421	7.731	0.958	0.013
	80-75	−2.737	3.800	3.850	5.493	0.999	0.001
	55-50	−3.962	−1.171	3.455	1.279	0.239	0.056
	35-30	2.730	2.115	2.127	7.404	0.935	0.011
Chlorophyll a + b [Chl a + b; mg g^{-1} FW]	100-95	1.784	8.704	1.848	9.359	0.196	0.097
	80-75	−9.369	8.378	1.645	1.185	0.998	0.007
	55-50	1.770	1.012	1.440	9.369	0.178	0.115
	35-30	2.646	−2.271	6.212	1.031	0.543	0.042
Chlorophyll stability index [CSI; %]	100-95	−2.130	−4.721	1.038	-8.799	0.987	1.030
	80-75	−9.369	4.732	9.292	1.185	0.998	0.377
	55-50	1.617	2.757	8.135	9.412	0.180	6.520
	35-30	−1.202	−3.556	3.299	8.110	0.922	1.089
Catalase activity	100-95	1.249	6.788	4.762	1.011	0.889	0.382

[CAT; nmol g^{-1} FW]	80-75	1.625	1.431	4.547	9.407	0.270	2.702
	55-50	1.613	2.043	4.164	9.413	0.193	4.164
	35-30	1.618	3.637	2.984	9.412	0.207	7.829
	100-95	9.652	1.697	4.424	5.042	0.985	0.346
Proline content	80-75	−1.737	1.485	6.356	7.668	0.995	2.061
[μmol g^{-1} FW]	55-50	−4.268	2.341	1.487	8.421	0.993	3.870
	35-30	1.264	3.842	1.921	6.522	0.995	5.133
	100-95	−3.772	1.461	2.774	9.536	0.480	1.159
Peroxidase activity	80-75	−1.237	−3.448	4.290	1.808	0.983	0.865
[POD; nmol g^{-1} FW]	55-50	2.558	5.277	3.781	8.537	0.992	0.894
	35-30	2.764	6.470	3.284	7.748	0.992	1.145
	100-95	−1.055	2.558	4.383	1.005	0.653	0.034
Malondialdehyde	80-75	−4.231	5.816	4.479	6.218	0.993	0.083
content [nmol g^{-1} FW]	55-50	3.778	1.073	7.179	−2.637	0.995	0.097
	35-30	2.239	1.818	9.417	6.558	0.997	0.199
	100-95	−3.140	1.099	2.210	7.866	0.965	0.389
Superoxide dismutase	80-75	2.243	4.611	2.060	2.178	0.231	2.264
[SOD: U mg^{-1} FW]	55-50	−3.075	2.766	1.906	7.578	0.999	0.113
	35-30	2.698	1.556	1.536	8.521	0.435	3.734
	100-95	−8.777	6.908	7.076	1.923	0.953	0.050
Silicon content	80-75	3.058	1.765	2.836	4.761	0.994	0.213
[Si; mg g^{-1} FW]	55-50	3.208	7.708	1.792	4.996	0.993	0.109
	35-30	3.275	6.230	1.526	5.088	0.999	0.023

Note: α, β, λ and ω are model constants, r = coefficient of determination and S = standard error.
